# Uncovering the Epigenetic Marks Involved in Mediating Salt Stress Tolerance in Plants

**DOI:** 10.3389/fgene.2022.811732

**Published:** 2022-04-12

**Authors:** Garima Singroha, Satish Kumar, Om Prakash Gupta, Gyanandra Pratap Singh, Pradeep Sharma

**Affiliations:** Indian Council of Agricultural Research-Indian Institute of Wheat and Barley Research, Karnal, India

**Keywords:** epigenetic modifications, DNA methylation, salt stress, RNA directed DNA methylatio, histone acetylation

## Abstract

The toxic effects of salinity on agricultural productivity necessitate development of salt stress tolerance in food crops in order to meet the escalating demands. Plants use sophisticated epigenetic systems to fine-tune their responses to environmental cues. Epigenetics is the study of heritable, covalent modifications of DNA and histone proteins that regulate gene expression without altering the underlying nucleotide sequence and consequently modify the phenotype. Epigenetic processes such as covalent changes in DNA, histone modification, histone variants, and certain non-coding RNAs (ncRNA) influence chromatin architecture to regulate its accessibility to the transcriptional machinery. Under salt stress conditions, there is a high frequency of hypermethylation at promoter located CpG sites. Salt stress results in the accumulation of active histones marks like H3K9K14Ac and H3K4me3 and the downfall of repressive histone marks such as H3K9me2 and H3K27me3 on salt-tolerance genes. Similarly, the H2A.Z variant of H2A histone is reported to be down regulated under salt stress conditions. A thorough understanding of the plasticity provided by epigenetic regulation enables a modern approach to genetic modification of salt-resistant cultivars. In this review, we summarize recent developments in understanding the epigenetic mechanisms, particularly those that may play a governing role in the designing of climate smart crops in response to salt stress.

## 1 Introduction

Unpredictable climatic conditions render plants suffer from an array of abiotic stress factors. Soil salinity is a key stressor impeding crop productivity and affects an area of more than one billion hectares all over the world and these numbers are constantly growing (FAO and ITPS, 2015).

**FIGURE 1:**
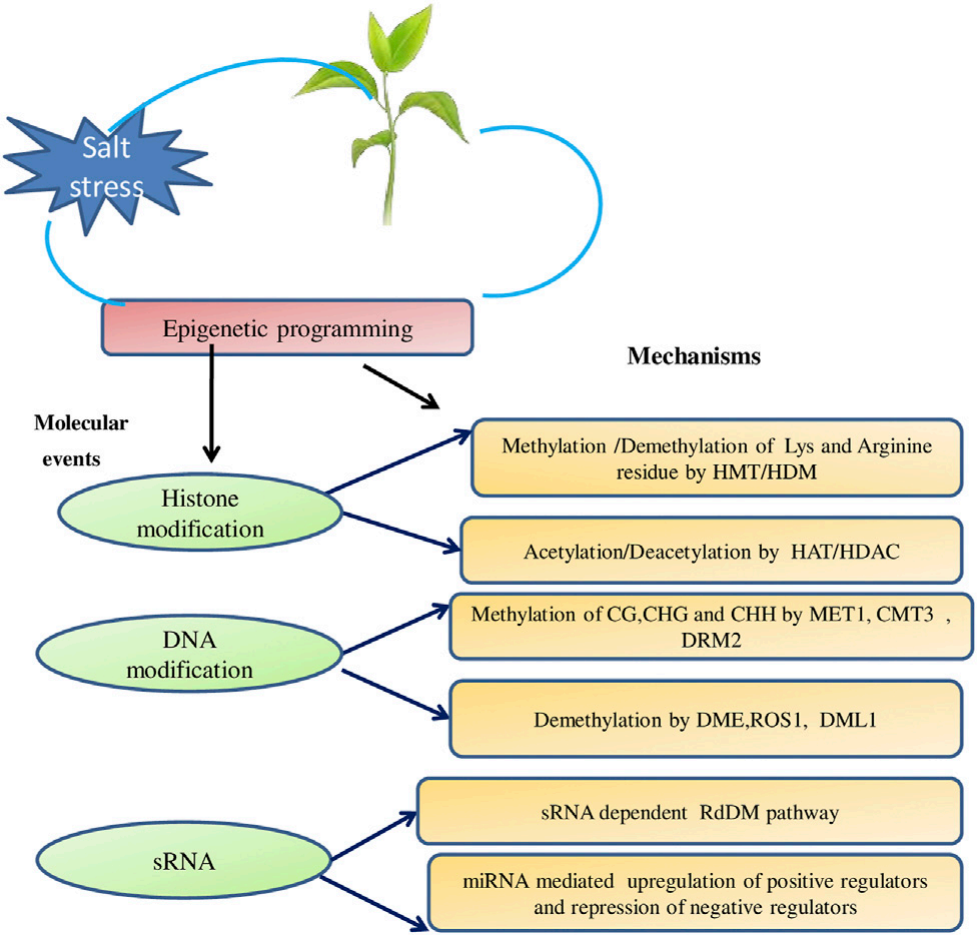
Schematic representation of epigenetics re-programming in plants exposed to salinity stress at three level, i.e., DNA modifications, Histone modifications and small RNAs.

At molecular level, plants respond to an environmental stress by implementing dynamic changes in gene expression and reprogramming the plant physiology (Lämke and Bäurle, 2017; Luo and He, 2020). In the last two decades, transcriptional responses have been explored to uncover the specific signaling pathways involved in salt stress responses and to distinguish the individual regulatory proteins and their targets. The chromatin architecture in eukaryotes is very dynamic and is modified in response to environmental stimulus. The transcriptional regulation of gene expression can be better apprehended by unveiling the underlying structural context. The regulation of gene expression by modulating chromatin architecture has been termed as epigenetics and is an essential mechanism for biological phenomena, including developmental programming, expression of genes, genome stability and small RNA-mediated regulation, and so forth (Chang et al., 2020). Epigenetic changes are changes in the DNA backbone independent of changes in its sequence and are decisive for plant life cycle (Duan et al., 2018). Important Epigenetic components are histone modification, histone variants, DNA methylation, and some noncoding RNAs (ncRNA) (Figure 1). These modifications demonstrate an overall impact on chromatin organization and sway its availability to the transcriptional machinery and hence act as a benchmark in regulating gene expression (Crisp et al., 2016; Saroha et al., 2017; Duan et al., 2018; Singroha and Sharma, 2019).

Methylation of DNA is the most extensively investigated epigenetic modification and includes the insertion of a methyl group at 5′ position on cytosine bases (called 5-methylcytosine or 5mC) or 6′ position of the adenine bases (called N6-methyladenine or 6 mA) (Liang et al., 2018; Zhang et al., 2018). DNA methylation is associated with numerous processes vital for plant growth and acclimatization to stress (Zhang et al., 2018). Several authors demonstrated a perturbation in methylation patterns and thus altered gene expression under saline conditions (Li et al., 2014; Wang B et al., 2015; Konate et al., 2018).

In order to counter unfavorable environmental conditions, histone protein sustain some modifications at their N′ termini to modulate the gene expression for better survival. It has now been documented that histone acetylation and methylation are vital epigenetic marks in fine tuning gene expression under unfavorable conditions (Xie et al., 2015a). H3K4me3 and H3K27me3 are generally viewed as a pair of the opponent markers for enhancing or diminishing the expression of marked genes all the way through environmental changes (Zhang et al., 2009). Apart from histone modification and methylation of DNA, histone variants also impact chromatin dynamics. On account of differences in amino acid sequence and structure each histone protein is defined by several variants. Different histone variants display varying affinities with DNA and other histone protein, which imparts them the capacity to modify the state of chromatin compaction and attract regulatory protein complexes. These epigenetic changes together govern the accessibility of DNA to transcriptional machinery and consequently influence gene expression under diverse stress conditions. The modern approach to genetic improvement of crops for environmental stress resilience seek to enhance stress tolerance and involves comprehensive knowledge of its interconnections and flexibility in the expression of epigenetic regulation (Rodríguez López and Wilkinson, 2015). Therefore, epigenetic determinants have attracted plant breeder’s interest since they are determinant of trans-generational phenotypic plasticity in plants under grueling environments. Hence, epigenetics play a very significant role in comprehending the complex mechanisms underpinning physical stress response and adaptability (Varotto et al., 2020). In this study, we have analyzed the current knowledge that connects the epigenetic and the transcriptional responses of plants under saline conditions, which might be essential for improving agricultural adaptability and reproducing climate smart crops.

## 2 DNA Methylation

Plant DNA methylation is referred to as N6-methyladenine (6 mA) or 5-methylcytosine (5 mC) (Zhang et al., 2018). However, in context of salinity 6 mA still remains enfolded and most of the reports acknowledge 5mC under salt stress. The 5mC is usually seen in all three sets of plant sequences: symmetrical CG and CHG together with asymmetrical CHH (where H = A, T or C) (Kumar et al., 2018). The methylation at different sequence contexts is catalyzed by sequence-specific methylases viz. CG methylation depends on MET1 (methyltransferase 1), CHG methylation requires DRM2 (domains rearranged methyltransferase 2) or CMT2 (chromomethylase 2) and CMT3 (chromomethylase 3) are vital for CHH methylation (Duan et al., 2018). The base excision pathway is one of the DNA repair pathways that can undo methylation state of a DNA and involves participation of DML2 (demeter-like 2), dme (demeter), ros1 (reprssor of transcriptional silencing 1) and five methylcytosine DNA glycosylase/DNA demethylase enzyme (Zhang et al., 2018; Liu and Lang, 2019).

Methylation of the promoter region has been generally associated with transcriptional repression while gene methylation activates transcription in *Arabidopsis thaliana*. Salt stress has been shown to affect methylation in different ways in different plant species and modify gene expression (Kinoshita and Seki, 2014; Voigt et al., 2015; Banerjee et al., 2017). Konate et al. (2018) observed increased DNA methylation in *Hordeum vulgaris* leaves as compared to roots and claimed that salt-induced methylation is organ-specific. Chen et al. (2019) observed that 61.2% of CGs, 39.7% of CHG, and 3.2% of CHHs were methylated under salt stress in *Glycine max* roots which represent significantly lower methylation compared to control.

**FIGURE 2:**
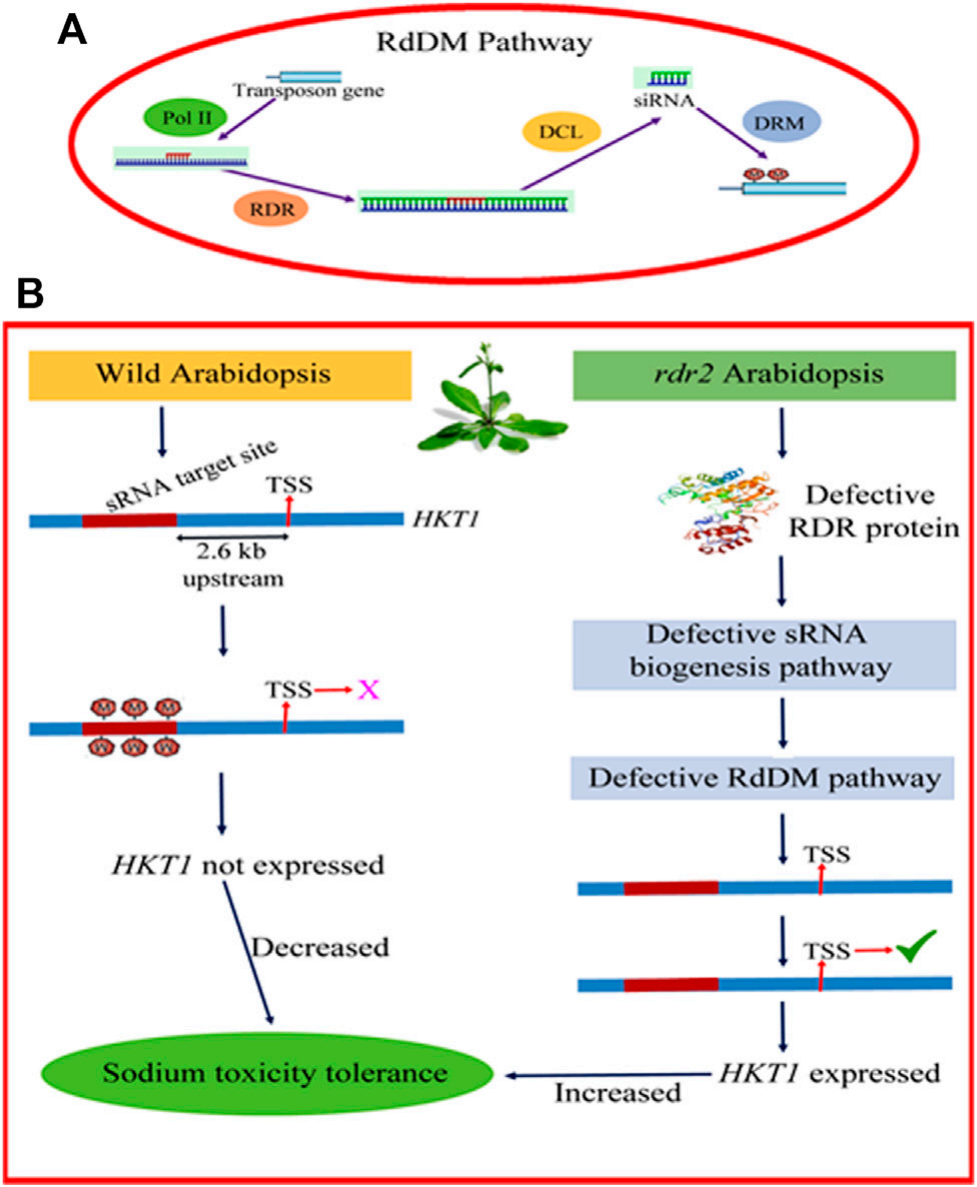
**(A)** RDR-dependent RdDM pathway. This pathway provides a means to establish RNA–directed DNA methylation (RdDM) and eventually ensure stable transcriptional gene silencing (TGS) **(B)** Role of RDR-dependent RdDM pathway in regulating the methylation landscape of *HKT1* gene in Arabidopsis.

More often, salt-induced DNA methylation occurs inside or in close proximity to already identified stress-responsive genes (Karan et al., 2012; Wang B et al., 2015; Wang B et al., 2015). The expression of stress responsive genes is influenced by transposable element insertions in their upstream regions. Shahid (2020) reported increased methylation at CHH and CHG context in Miniature Inverted Repeat Transposable Elements in *OsHKT1;5* gene under salt stress. He observed role of methylation in regulation of *OsHKT1;5* gene (a major salt tolerance gene in rice that encode Na^+^ transporter for exclusion of Na^+^ from leaves and is important for Na^+^/K^+^ homeostasis under salt stress) and thus endowing salt tolerance (Figure 2B). High frequency of hypermethylation in the promoter located CpG sites has also been reported under salt stress conditions (Kumar et al., 2017; Ashapkin et al., 2020; Skorupa et al., 2021).

Owing to its heritable nature, any DNA methylation changes caused by environmental perturbations in plants have the prospect to be perpetuated and disseminated to future generations. This permits stress elicited methylation changes to proceed as a “memory” and help prime the plant or its progeny to counter more competently to the stress if re-exposed (Chaudhary et al., 2021).

## 3 Histone Modifications

Histones are basic proteins consisting majorly of lysine and arginine residues that lay down the foundation of nucleosomal chromatin organization (Zhou et al., 2013). The N’ termini of histone proteins, known as histone tails are the sites of covalent modifications such as acetylation, methylation, ubiquitination and phosphorylation. This covalent modification imparts different effects depending on the amino acid residue being modified and thus alters the genes activity (Banerjee et al., 2017). Indeed, studies in different plant species have demonstrated that histone modification is imperative to regulating gene expression under salt stress (Song et al., 2012; Li et al., 2014; Shen et al., 2014). Paul et al. (2017) reported differential regulation of *OsBZ8* gene expression due to significant differences in chromatin modification between *Oryza sativa* varieties IR64 and Nanabokra under salt stress. It has been demonstrated that tempering histone proteins provide an epigenetic molecular apparatus for priming plants to salt stress via the modulation of crucial salt responsive genes perpetuated throughout vegetative growth (Pikaard and Mittelsten Scheid, 2014).

### 3.1 Histone Acetylation

A negatively charged acetyl moiety on H3 and H4 histones serve to reduce the affinity between DNA and histone protein, enhancing DNA’s accessibility to the transcriptional machinery (Onufriev and Schiessel, 2019). Acetylation of Lys residue 9 of histone H3 (H3K9ac) is largely investigated covalent modification and acts as new layers of supervision to cope with abiotic environmental stress through modulation of key regulatory factors (Zheng et al., 2016; Li et al., 2017; Ueda et al., 2017). Histone acetylation is frequently related with increased gene expression while deacetylation is associated with transcriptional repression (Zheng et al., 2016). Histone acetyl transferase (HATs) and histone deacetylases (HDAC) are the key enzymes that offer powerful transcriptional control mechanisms by catalyzing the addition and removal of an acetyl moiety respectively (Zhou et al., 2017; Kim et al., 2018).

**FIGURE 3:**
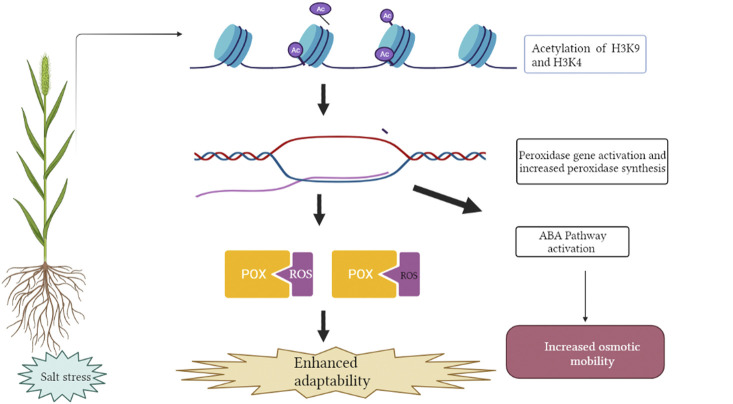
Deposition of acetylation at H3K4 and H3K9 position leads to activation of salt responsive POX gene encoding peroxidase enzyme. Increased expression of peroxidase gene has been associated with activation of the ABA pathway (Absicsic acid) and antioxidant enzymes, resulting in lower ROS (Reactive Oxygen Species) accumulation and increased levels of osmotic metabolites. This figure was created using https://biorender.com.

Salt induced histone acetylation is linked with transcriptional activation of salt stress responsive genes reported in the case of *Nicotiana tabacum* (Sokol et al., 2007), *Zea mays* (Li et al., 2014) and *Saccharomyces cerevisiae* (Magraner-Pardo et al., 2014). Yolcu et al. (2016) demonstrated deposition of active histone marks such as H3K9ac and H3K4ac on the peroxidase gene resulting in its activation in *Beta vulgaris* and *B. maritime* (Figure 3). Increased expression of peroxidase gene has been linked with an activation of the ABA (abscisic acid) pathway and antioxidant enzymes, resulting in lower ROS (reactive oxygen species) accumulation and increased levels of osmotic metabolites therefore, augmenting salt tolerance (Su et al., 2020). Sako et al. (2016) reported that increased histone acetylation of *AtSOS1* and *AtSOS3* play an important role in salinity stress. Increased acetylation contributes to open a more relaxed chromatin confirmation ready for transcription. *TaHAG* (histone acetyltransferase) mediated H3 acetylation of polyploidy wheat genes involved in ROS production has been reported to up-regulate transcriptional changes of these genes in response to salt stress (Zheng et al., 2021). This gene in wheat and other crops can be manipulated as a potential target for salt tolerance improvement.

#### 3.1.1 Role of HATs in Salinity Stress

The *Arabidopsis thaliana* genome contains four HAT (Histone acetylase transferase) gene families encoded by 12 HAT genes (Earley et al., 2007). Under salt stress conditions, the expression of cell wall related genes *ZmEXPANSIN B2* and ZmXYLOGLUCAN endotransglucosylase/hydrolase1) are up-regulated due to increased H3K9 acetylation at both the promoter and coding regions of genes. The increased acetylation of these genes is attributed to increased mRNA expression of two HAT genes (*ZmHATB and ZmGCN5*) under salt stress (Li et al., 2014). These observations have been further supported in *Arabidopsis*, where H3K9/K14 acetylation resulted in elevated expression levels of GCN5 under salt conditions and activated chitinase-like (CTL) protein involved in cell wall biosynthesis and salt tolerance (Zheng et al., 2019).

Although some HAT gene expression levels are shown to increase H4K5 acetylation during salt stress conditions, certain Histone deacetylases respond negatively to the salt stress resistance. Similarly *OsHDA1* was reported to negatively affect the transcriptional activation of *OsSOS1* in rice (Cheng et al., 2018). Zheng et al. (2021) claimed *TaHAG1* (histone acetyl transferase) to play decisive role in strengthening the salt tolerance in bread wheat. Further understanding the defined mechanisms by which HATs activities are modulated will offer new insight into the complex network regulating plant adaptation and tolerance to stress.

#### 3.1.2 Role of Histone Deacetylases in Salinity Stress

Under favorable conditions, the repressive chromatin state of stress responsive genes is preserved by Histone deacetylases to keep gene transcripts at low levels. Histone deacetylases are involved in removing acetyl groups. Plants contain three families of Histone deacetylase proteins, i.e., i) Reduced potassium dependency 3 (RPD3)-like, ii) Silent Information Regulator 2 (SIRT) and iii) HD-tuins. The three Histone deacetylase families in the *A. thaliana* genome are encoded by 18 genes. Studies documented that upon exposure to abiotic stress, histone deacetylase genes display diversified responses and play a crucial role in how plants behave under such conditions (Asensi-Fabado et al., 2017; Kim et al., 2018).

Histone deacetylase over-expression in transgenic poplar plants reduced tolerance under salt stress (Ma et al., 2019a). Histone deacetylase9 constitutes a core histone deacetylase complex with PWR (POWERDRESS) and HOS15 (HIGH EXPRESSION OF OSMOTICALLY RESPONSIVE GENES), that binds to and directly represses many abiotic/biotic stress-responsive genes, including ethylene response factor (ERF) (ERF4/5/6/11), salt tolerance zinc finger (STZ), and kinase 2 (KIN2) genes, by modulating both histone acetylation (H3K27ac/H3K36ac/H3K56ac, H3.3K27/36ac andH4ac) and methylation (H3K9me2 and H3.1K36me2) (Mayer et al., 2019). Similarly *OsHDA1* was reported to negatively affect the transcriptional activation of *OsSOS1* in rice (Cheng et al., 2018). The OsHDA1 (histone deacetylase HDA1) is involved in the suppression of *s*alt overly sensitive1 (*SOS1*) and late embryogenesis abundant protein1 (*LEA1*) genes, which are essential for salt tolerance in rice, by decreasing H3 acetylation in the promoter regions of *LEA1* and *SOS1* genes (Cheng et al., 2018). In *Arabidopsis* class I (HDA19) family histone deacetylases are implicated in positive salinity responses and class II (HDA5/14/15/18) reduced potassium dependency3 (RPD3) histone deacetylases are involved in negative salinity responses (Ueda et al., 2017; Ueda et al., 2019). In *Hibiscus cannabinus,* HcHDA2, HcSRT2, HcHDA6, HcHDA8, HcHDA9, HcHDA19, and the levels of acetylation at H3K9ac, H3K27ac, and H4K5ac under salt stress conditions have been shown to be up-regulated (Wei et al., 2019). Similarly, HDA710/OsHDAC2, an HDA RPD3/HDA1 family member, contributes to controlling the rice salt stress genes by altering levels of H4 acetylation in their promoters. It regulates the acetylation at H4K5 and H4K16 under normal conditions. The accumulation of HDA710 transcripts under salt stress was considerably enhanced (Ullah et al., 2020). It is fascinating to break down specific function of the diverse HDACs in stress tolerance, genome-wide recognition of their target genes and investigation of alteration in histone acetylation at these genes under stress conditions. Moreover, how HDACs react to stress signaling to manage histone acetylation and expression of specific genes remains elusive.

### 3.2 Histone Methylation

Contrary to acetylation, histone methylation does not affect the electrostatic properties of histone proteins but it increases the hydrophobicity by changing intra or intermolecular interactions and may create novel binding sites for other proteins (Liu et al., 2010). Methyl group at Arg residue is added by Arg methyltransferases (PRMTs) while addition of methyl group at Lys residues is catalyzed by histone Lys methyltransferases (HKMTs). Two Arg methylation sites (H3R17 and H4R3) and five Lys methylation sites (H3K4, H3K9, H3K27, H3K36, and H4K20) have thus far been identified in plants (Liu et al., 2010). In *Glycine* max and *A. thaliana*, salt stress has been reported to increase methylation at fourth lysine of H3 (H3K4me3) and decrease histone H3 lysine 9 dimethylation and/or decreases histone H3 lysine 9 dimethylation (H3K9me2) associated with salt responsive genes (Bilichak et al., 2012; Song et al., 2012). Histone methylation in *Arabidosis* represent repressive (H4R3me2, H3K9me2/3, and H3K27me3) and active marks (H4R3me2, H3K4me3, and H3K36me2/3 (Liu et al., 2016). The presence or absence of methylation of Lys and/or Arg amino acids in histones alters their association with reader proteins, leading to modifications in chromatin structure that result in either transcriptional repression or activation (Teperino et al., 2010). Similarly, DNA methylation of H3 at 4th and 27th lysine in castor and rice crop plants has been demonstrated to regulate transcription of the critical salinity-response regulator (Karan et al., 2012; Han et al., 2020). Transcription of RSM1 (RADIALIS LIKE SANT-an MYB TF and key salt response regulator in salt signaling) has been reported to be guided by methylation at H3K4 and H3K27 in castor (Han et al., 2020). In the recent past it was found that the H3K4me0/1/2 code reader (GmPHD6) could specifically regulate the transcription of some salt-tolerance genes in *Glycine* max (Wei et al., 2017). Variation in methylation level at H3K4me3 and H3K27me3 has been reported to display differential expression level of salt responsive *OsBZ8* gene in rice varieties Nonabokra (salt tolerant) and IR64 (salt sensitive) (Paul et al., 2017). These observations evidently established important role of epigenetic marks H3K4me3 and H3K27me3 in regulating salt stress responsive genes and imparting salt tolerance. Furthermore, *JMJ15* gene (coding for H3K4 demethylase) over expression in *A. thaliana* under salt stress radically improved salt tolerance (Shen et al., 2014). The effects of histone methylation events vary depending on the site of the modification. For example, tri-methylation of the fourth lysine of H3 (H3K4me3) is an active mark for gene expression, and tri-methylation in the 27th lysine of H3 (H3K27me3) is a repressive mark of facultative heterochromatin (Doyle and Amasino, 2009). Although changes in histone modifications can be correlated with gene activity, the molecular mechanisms through which the chemical modifications influence chromosomal structure and the accessibility of transcription factors are still not fully understood. These relationships between the alteration of histone modifications and gene activity are highly conserved from yeast to human, and also in plants.

The histone methylation and acetylation have been extensively investigated in different plant species under salt stress conditions. Investigations deciphering other histone modifications may enrich our knowledge about other important epigenetic marks and their exploitation for breeding climate smart crops.

## 4 Histone Variants

Of the various factors influencing chromatin dynamics and accessibility histone variants are also among the important ones that participate in modulating gene expression. Many species have been shown to encode numerous genes for core histone proteins, which are quite similar in amino acid sequence. Like histone proteins histone variants have also been shown to be differently expressed in *Oryza sativa* and *A. thaliana* (Talbert and Henikoff, 2021). In *A. thaliana*, 11 genes for H2B have been discovered, 13 for H2A, and 15 for H3 (Probst et al., 2020). The discovery that histone variant expression is tissue and developmental stage specific suggests that histone variations have particular functions in altering structural and functional properties of chromatin.

Histone variations that are replication-independent and replication-dependent can substitute for each other and are deliberately positioned within the genome. Each of the four histones (H2A, H2B, H3, and H1) have distinct variants. H2A is the most widely investigated histone and consists of H2A, H2A.Bbd, H2A.X, and H2A.Z variants (Bonisch and Hake, 2012). Similarly 14 variants of H4 (Siegel et al., 2009; Moosmann et al., 2011; Bonisch and Hake, 2012) and two different isoforms of H2A known as H2A.Z.1 and H2A.Z.2 displaying specific functions (differing in only three amino acids) have been reported (Coon et al., 2005; Eirín-López et al., 2009; Talbert et al., 2012). The expression of the H2A.Z variant of H2A histone has been diminished in *O. sativa* and *A. thaliana* during salt or other stress (Nguyen and Cheong, 2018; Zahraeifard et al., 2018). H2A.Z has been portrayed as a crucial thermosensor (Kumar and Wigge, 2010) during stress response. H2A.W predominantly found in heterochromatin is engaged in stress induced chromatin decondensation. In *A. thaliana* replacement of H3.3 has been shown to be correlated with transcriptional process and declining H3.3 brings down transcription of stress responsive genes (Wollmann et al., 2017). Accumulation of H3.3 avert H1 histone from acquiring its position at gene body to assist DNA methylation which further alienate deposition of H2A.Z (Zilberman et al., 2008; Wollman et al., 2017). This explains why H3.3 is indispensable for stress responsive gene expression. This aspect of chromatin modification is however not much explored yet and offer exciting possibilities to understand the role of histone variants at different growth and development stages in response to stress. Deposition of histone variations under stress gives the possible way to connect environmental cues to transcription downstream reactions. More investigations are required to define how it generates epigenetic memory clearly.

## 5 Plant microRNAs and Long Non Coding RNAs: Key Epigenetic Regulators

Plants adopt *de novo* DNA methylation and gene silencing (transcriptional) using 24- nucleotide small-interfering RNAs and long non-coding RNAs in the RNA-directed DNA methylation process (Kovalchuk, 2016). RNA dependent DNA methylation (RdDM) is the only system in plants that can introduce DNA methylation to cytosines irrespective of the sequence context (Magraner-Pardo et al., 2014) (Figure 2A). This pathway helps plants in surviving under adverse environmental conditions like salt stress (Fortes and Gallusci, 2017). Under saline conditions RdDM becomes down-regulated and elicit the expression of transcription factors central for salt stress tolerance (Xie et al., 2015a). The plant microRNAs (miRNA) are 20–24 nt, non-coding RNA species that have been portrayed as tiny yet potent regulators of gene expression in plants as well as animals. These miRNAs are either positively regulated by stress, where they enhance the repression of the genes serving as negative regulators of stress tolerance or negatively regulated where the target is positive regulator of stress causing more accumulation of gene product (Sunkar et al., 2007; Singroha et al., 2021). The biogenesis of miRNAs has been reviewed by Singroha et al. (2021). Most of the miRNAs responsive to salt stress directly regulate transcription factors. miR164a/b/c/d/and miR1661m identified from *Zea mays* have been shown to target *MYB, NAC* and homeodomain-leucine zipper protein (HD-ZIP) transcription factors under salt stress (Ding et al., 2009). It has also been observed that miRNA exhibit species specific behavior in response to salt stress. For instance the expression of miR156 was induced under salt stress in *A. thaliana* while diminished in *Z. mays* (Liu et al., 2008; Ding et al., 2009). In the same way expression of miR396 was up-regulated in *A. thaliana* and *Z. mays* upon salt treatment but diminished in *O. sativa* (Liu et al., 2008; Ding et al., 2009).

*MYB74* (a member of the R2R3-MYB gene family) is transcriptionally regulated mainly by RdDM pathway under salt stress in *A. thaliana*. 24-nt siRNAs (small interfering RNA) target a region approximately 500bp upstream of the transcription start site of *MYB74,* which is heavily methylated. Levels of DNA methylation in this region were significantly diminished in wild type plants under salt stress, whereas no changes were observed in RdDM mutants. These observations suggest that changes in the levels of the five 24-nt siRNAs regulate the *MYB74* transcription factor via RdDM under salt stress conditions (Xu et al., 2015). The salt-tolerant regulation of MYB transcription factors involves ABA signaling pathway and other signal transduction pathways in plants. Salt stress subjected plants exhibited significantly increased ABA content that can induce proline accumulation in plants, and enhance the activity of related protective enzyme and up-regulation of related stress responsive genes (Schmidt et al., 2013). The investigations made in this area have tried to extend our understanding of non-coding RNAs functional processes for salt stress in *A. thaliana* (Qin et al., 2017), *H. vulgare* (Karlik and Gozukirmizi, 2018), cotton (Zhang et al., 2019a), *Spirodela polirhiza* (Fu et al., 2020) and sorghum (Sun et al., 2020).

Under salt stress conditions *Z. mays* displayed down-regulation of miR-250, miR-205, miR-330 and miR-17 in leaves and roots (Fu et al., 2017). Down-regulation of these miRNAs enhanced the expression of their targets viz. *casein kinase II*, *GPX*, *P5CS*, *IF-1* and some other genes essential for better survival of the plant under saline conditions. This is how miRNAs regulate gene expression under stress conditions and help plants in their survival under harsh environmental conditions. Apart from 24 nt long miRNAs, the long non-coding RNAs abbreviated as lncRNAs have also been defined as riboregulators longer than 200 bp (Kapranov et al., 2007). They also regulate gene expression under stress conditions through transcriptional or post transcriptional silencing. Chen and associates (2019) identified 3030 long intergenic non-coding RNAs in *Glycine max* roots under salt stress conditions. For example, the long non-coding RNA NPC60 expression was escalated 100 times under salt stress condition. Similarly salt treatments enhanced levels of long non-coding RNA973 in cotton (Zhang et al., 2019b). The over expression of lncRNA973 displayed high salt tolerance, which modulates cotton salt genes expression. Ma et al. (2019a) demonstrated tissue, and species specific expression of long non-coding RNA in Poplar species under different salt stress conditions. A list of plant small and long non-coding RNAs expressed in response to salt stress is provided in Table 1.

**TABLE 1:** Long non coding RNAs/miRNAs involved in imparting salt tolerance.

S.N.	lncRNA/miRNA	Plant Species	Characteristics	References
1	ThSAIR6	*Tamarix hispida*	Decreased the contents of H_2_O_2_ and enhanced activity of anti-oxidative enzymes	Xu et al. (2021)
2	AtR8lncRNA	*Arabidopsis thaliana*	Regulate seed germination in response to salt	Zhang et al. (2020)
3	LncRNA973	*Gossypium hirsutum*	Increased expression resulted into increased salt tolerance	Zhang et al. (2019)
4	Pal_00132209	*Populus alba*	Affect fucosyltransferase or NAC3 and regulates growth under salt stress	Ma et al. (2019b)
5	Pal _00184400	*Populus alba*	HKT1 and show differential expression in xylem	Ma et al. (2019b)
6	lnc_388, lnc_973, lnc_253	*Gossypium hirsutum*	Regulates tolerance to salt stress	Deng et al. (2018)
7	DRIR (Drought Induced long non coding RNA)	*Arabidopsis thaliana*	Regulates ABA mediated responses to both salt and drought	Qin et al. (2017)
8	*TCONS_00116877*	*Medicago truncatula*	Regulates oxidative stress under salt conditions	Wang et al. (2015)
9	*TCONS_00046739*	*Medicago truncatula*	Regulates cytochrome P450 under salt stress	Wang et al. (2015)
10	miR156, miR398	*Solanum lycopersicum*	Increased expression levels imparted salt tolerance	Cakir et al. (2021)
11	nta-miR156a_R + 3, farmiR159_L + 2_1ss22T, mes-MIR319e- p5_2ss12GC19 GA	*Ipomoea batatas*	tissue specific expression under salt stress	Yang et al. (2020)
12	miR26, miR05, miR20, miR31, miR11, miR28, miR15, miR14, miR32, miR09, miR22, miR33, miR19, miR24	*Pennisetum glaucum*	Shows altered expression under salinity	Shinde et al. (2020)
13	miR172, miR319, miR408, miR2590	*Medicago sativa*	Regulates gene associated with salt tolerance	Ma et al. (2019b)
14	TaemiR408	*Triticum aestivum*	Overexpresion resulted in enhanced salt tolerance	Bai et al. (2018)
15	miR164s, mir-36	*Zea mays*	up-regulated in leaves under salt treatment	Fu et al. (2017)
16	osa-miR1878, osa- miR2863c	*Oryza sativa*	Upregulated under salt stress	Goswami et al. (2017)
17	miR171b, miR167f	*Oryza sativa*	Promotes better adaptability to salt	Parmar et al. (2020)
18	sly-miR156e-5p, slymiRn23b, slymiRn50a	*Solanum pimpinellifolium*	Involved in stress related pathways	Zhao et al. (2017)
19	miR172	*Glycine max*	Improves salt tolerance	Pan et al. (2016)
20	miRNVL5	*Gossypium hirsutum*	Regulation of plant stress to salt	Gao et al. (2016)
21	miR-395	*Cucumis sativus*	Up-regulated and regulates ATP sulfurylase	Li et al. (2016)
22	miR156/157, miR158, miR166, miR168 and miR408	*Raphanus sativus*	Expression was upregulated significantly	Sun et al. (2015)
23	miR-160	*Gossypium raimondii*	Up-regulated under salt stress and control Auxin response factor (ARF)	Xie et al. (2015)

## 6 Conclusion and Future Outlook

Many findings have emphasized epigenetic regulations as powerful mechanisms for regulating the implications of salt stress on plants and provide an excellent foundation for development of salt-tolerant crop plants. In plants susceptible to salt stress, epigenetic controls are associated with the stringent control of gene expression. Epigenetic marks on stress-induced genes dynamically affect the accessibility of chromatin and the expression of those genes. The different regulatory mechanisms for abiotic stress responses might involve epigenetic alterations such as methylation, histone changes, chromatin remodelling, histone variants and lncRnAs.

The critical role of epigenetic modifications in regulating gene expression and their ability to transfer to the next generation makes them a unique adaptation tool for plants. The phenotypic plasticity caused by epigenetic variation, which in turn, is through changes in gene expression, will affect fitness and eventually natural selection in plants. Unlike classic DNA sequence mutations, epimutations can happen at much shorter times, and even though they are stable, they are primarily reversible, making them a perfect tool for a quick emergency response to unpredictable environmental stresses. It must also be highlighted that epigenetic changes are typically dependent on the underlying genetic variation, and these two factors must be addressed concurrently. Future study is required to better understand the epigenetic mechanisms behind chromatin changes and the resulting transcriptional regulation that impacts plant responses to environmental stresses. More study on the mechanism of hereditary stress memory is also required.
